# Transposition of Great Arteries with Intramural Coronary Artery: Experience with a Modified Surgical Technique

**DOI:** 10.5935/1678-9741.20160003

**Published:** 2016

**Authors:** Amit Mishra, Anil Jain, Manish Hinduja, Vivek Wadhawa, Ramesh Patel, Nikunj Vaidhya, Dayesh Rodricks, Hardik Patel

**Affiliations:** 1 Department of Cardiovascular and Thoracic Surgery, U.N. Mehta Institute of Cardiology and Research Center (Affiliated to B. J. Medical College), Civil Hospital Campus, Asarwa, India.; 2 Department of Cardiac Anesthesia, U.N. Mehta Institute of Cardiology and Research Center (Affiliated to B. J. Medical College), Civil Hospital Campus, Asarwa, India.; 3 Department of Perfusion, U.N. Mehta Institute of Cardiology and Research Center (Affiliated to B. J. Medical College), Civil Hospital Campus, Asarwa, India.; 4 Medical Officer, U.N. Mehta Institute of Cardiology and Research Center (Affiliated to B. J. Medical College), Civil Hospital Campus, Asarwa, India.

**Keywords:** Heart Defects, Congenital, Surgery, Transposition of Great Vessels, Coronary Artery Disease, Ventricular Function

## Abstract

**Objective:**

Transposition of the great arteries is a common congenital heart disease. Arterial switch is the gold standard operation for this complex heart disease. Arterial switch operation in the presence of intramural coronary artery is surgically the most demanding even for the most experienced hands. We are presenting our experience with a modified technique for intramural coronary arteries in arterial switch operation.

**Methods:**

This prospective study involves 450 patients undergoing arterial switch operation at our institute from April 2006 to December 2013 (7.6 years). Eighteen patients underwent arterial switch operation with intramural coronary artery. The coronary patterns and technique used are detailed in the text.

**Results:**

The overall mortality found in the subgroup of 18 patients having intramural coronary artery was 16% (n=3). Our first patient had an accidental injury to the left coronary artery and died in the operating room. A seven-day old newborn died from intractable ventricular arrhythmia fifteen hours after surgery. Another patient who had multiple ventricular septal defects with type B arch interruption died from residual apical ventricular septal defect and sepsis on the eleventh postoperative day. The remainder of the patients are doing well, showing a median follow-up duration of 1235.34±815.26 days (range 369 - 2730).

**Conclusion:**

Transposition of the great arteries with intramural coronary artery is demanding in a subset of patients undergoing arterial switch operation. We believe our technique of coronary button dissection in the presence of intramural coronary arteries using coronary shunt is simple and can be a good addition to the surgeons' armamentarium.

**Table:** 

**Abbreviations, acronyms & symbols**	
ASO	= Arterial switch operation
CHDs	= Congenital heart defects
ECG	= Electrocardiogram
IVS	= Intact ventricular septum
TGA	= Transposition of the great arteries
VSD	= Ventricular septal defect

## INTRODUCTION

Transposition of the great arteries (TGA) is a congenital cardiac anomaly in which the aorta arises entirely or largely from the right ventricle and the pulmonary trunk arises entirely or largely from the left ventricle, also known as discordant ventriculoarterial connection^[1]^. It is the most frequent cyanotic heart disease diagnosed in newborns and it accounts for 5% to 7% of all congenital heart defects (CHDs), with a prevalence rate of 0.2 per 1000 live births. Arterial switch operation (ASO) is the operation of choice for TGA.

The term "intramural coronary artery" refers to the coronary patterns in which there is an intimate relationship between aortic and coronary arterial walls; histologically, the aortic and coronary medial walls are attached without interposed adventitia^[2]^. Intramural coronary arteries are rare in patients with normal ventriculoarterial connections, but they are proportionally more common in TGA, with a reported incidence of 3% to 5%. The intramural artery usually originates from the wrong sinus and runs between the great arteries. Sometimes the intramural artery originates from its normal sinus, but above the sinotubular junction. These abnormal coronary patterns might complicate coronary transfer during ASO^[2]^. We present our modified surgical technique with a prospective analysis of the data.

## METHODS

**Fig. 1:**
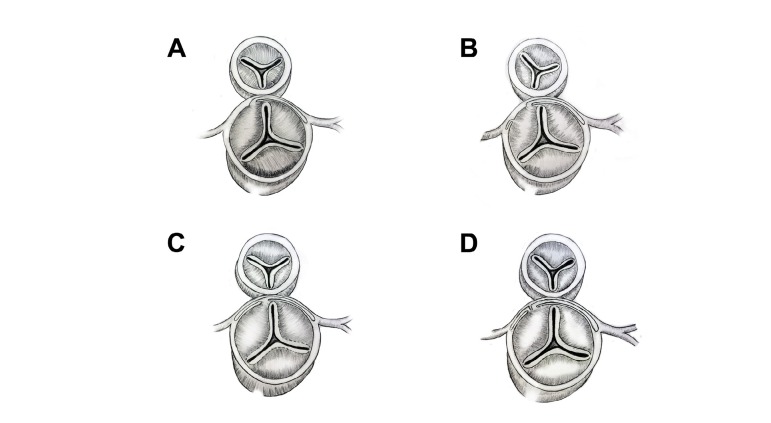
Intramural coronary artery pattern observed in eighteen patients.

This prospective study was conducted at U. N. Mehta Institute of Cardiology and Research Centre for 7.6 years. After receiving approval from the institutional ethics committee, a total of 450 patients who underwent ASO from April 2006 to December 2013 were included in the study (Table 1). All of the patients were diagnosed with two-dimensional echocardiography. Two hundred and seventy six patients had TGA with intact ventricular septum (IVS), 141 patients had TGA with ventricular septal defect (VSD) (six of them had multiple VSD), and 33 patients had Taussig-Bing anomaly (one had multiple VSD). Three patients in the TGA/ VSD group and two with Taussig-Bing anomaly had type-B arch interruption. Eighteen patients (Table 2) had intramural coronary artery (Figure 1), eleven of which were from the TGA/VSD group and seven from the TGA/IVS group (one was on prostaglandin infusion and two underwent balloon atrial septostomy on the 4^th^ and 6^th^ day of life). None of the patients from the Taussig-Bing anomaly group had intramural coronary artery. Similarly, none of the eighteen patients had preoperative diagnosis/suspicion of intramural coronary pattern. Eight patients with intramural coronary arteries had both left and right coronary arteries having intramural course arising from a single ostium from Sinus 2 (one of them had a separate opening with kissing edges). The patients with intramural course were operated on by a single surgeon. The technique of separation of coronaries into two buttons was used in all eighteen patients.

**Table 1:** Details of the patients who underwent arterial switch operations.

Total: 450 patients	
Diagnosis	Number of Patients (%)
Transposition of the Great Arteries with Intact Ventricular Septum	276 (61.33%)
Transposition of the Great Arteries with Ventricular Septal Defect	141 (31.33%)
Taussig-Bing anomaly	33 (7.3%)
Total: 18 (4%) Patients with Intramural Coronary Artery	
Transposition of the Great Arteries with Ventricular Septal Defect	11 (61.11%)
Transposition of the Great Arteries with Intact Ventricular Septum	7 (38.88%)

**Table 2:** Demographic details of patients with intramural coronary artery.

No	Age (days)	Sex	Weight	Diagnosis	Coronary Pattern	Great Artery Relationship	Outcome
1	45	M	3.1	D-TGA VSD	2 LRCX	Aorta Anterior to PA	Doing well on regular follow-up
2	38	M	3.0	D-TGA IVS	2 LRCX	Aorta Anterior to PA	Died due to accidental injury to left intramural coronary artery
3	42	F	3.0	D-TGA VSD	2 LRCX	Aorta Anterior to PA	Doing well on regular follow-up
4	62	M	3.4	D-TGA VSD	2 LRCX	Aorta Anterior to PA	Doing well on regular follow-up
5	25	F	3.1	D-TGA IVS	2 LRCX	Aorta Anterior to PA	Died from intractable ventricular arrhythmias fifteen hours after surgery
6	36	F	2.6	D-TGA VSD	2 LRCX	Aorta Anterior to PA	Doing well on regular follow-up
7	42	F	3.2	D-TGA, Multiple VSD & type B arch interuption	2 LRCX	Aorta Anterior to PA	Died secondary to sepsis and residual VSD
8	90	F	3.9	D-TGA VSD	2 LRCX	Aorta Anterior to PA	Doing well on regular follow-up
9	55	M	3.0	D-TGA VSD	2 LRCX	Aorta Anterior to PA	Doing well on regular follow-up
10	48	F	2.9	D-TGA VSD	2 LRCX	Aorta Right & Anterior to PA	Doing well on regular follow-up
11	42	F	3.2	D-TGA VSD	2 LRCX	Aorta Anterior to PA	Doing well on regular follow-up
12	38	M	3.2	D-TGA VSD	2 LRCX	Aorta Anterior to PA	Doing well on regular follow-up
13	27	M	3.1	D-TGA IVS	2 LRCX	Aorta Anterior to PA	Doing well on regular follow-up
14	19	F	2.7	D-TGA IVS	2 LRCX	Aorta Anterior to PA	Doing well on regular follow-up
15	33	F	2.4	D-TGA IVS	2 LRCX	Aorta Anterior to PA	Doing well on regular follow-up
16	27	F	2.2	D-TGA IVS	2 LRCX	Aorta Anterior to PA	Doing well on regular follow-up
17	18	M	2.4	D-TGA IVS	2 LRCX	Aorta Anterior to PA	Doing well on regular follow-up
18	44	F	3	D-TGA VSD	2 LRCX	Aorta Right & Anterior to PA	Doing well on regular follow-up

D-TGA=dextro transposition of the great arteries; IVS=intact ventricular septum; VSD=ventricular septum defect; LRCX=left-right circumflex

Morphologic and demographic characteristics of the patients with intramural coronary artery are given in Figure 1 and Table 3.

A- Intramural left coronary artery with normal origin of right coronary artery (9 patients).

B- Intramural left and right coronary artery with separate openings (1 patient).

C- Intramural left and right coronary arteries with single ostia (7 patients).

D- Intramural left and right coronary artery with kissing ostial openings (1 patient).

**Table 3:** Demographic details and surgical outcomes of patients with intramural coronary artery.

Variables	Mean±SD
Age (days)	40.61±I6.94
Weight (kg)	2.96±0.40
Height (cm)	49.66±3.08
Sex	M=7 F=ll
Mortality	3 (16.66%)
Survival duration (days)	Mean: 1245.94±836.7
	Median: 1788.5 (369 - 2920)

### Statistical analysis

The statistical calculations were performed using SPSS software v.20.0 (Chicago, IL, USA) Quantitative data were expressed as mean±SD whereas qualitative data were expressed as percentage. Survival statistics were performed using Kaplan-Meier survival curve.

### Technique (Figure 2)

**Fig. 2:**
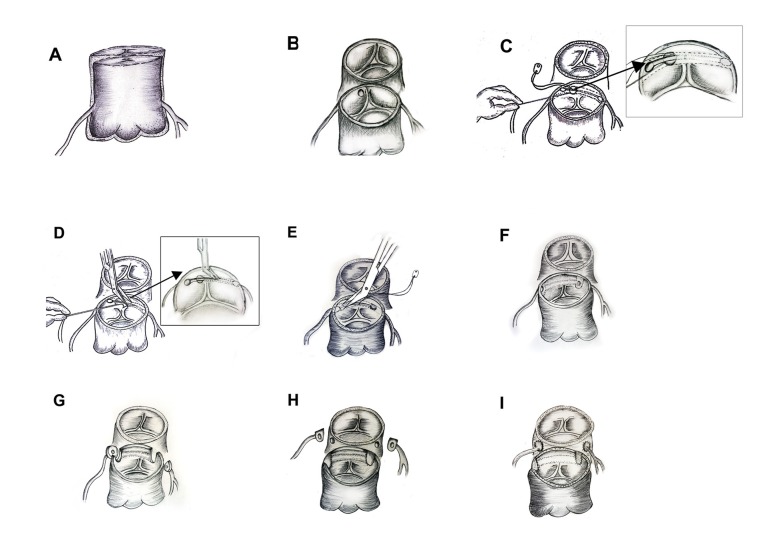
Anatomical patterns of the coronary arteries.

The standard technique of cardiopulmonary bypass and cardioplegic arrest was used (aortic and single right atrial cannulation for TGA with IVS and bicaval cannulation for the TGA/VSD group; multiple dose ST Thomas cardioplegia was used in the first nine patients and Del nido cardioplegia in the other nine). The aorta was transected at the level of the pulmonary artery bifurcation and it was inspected from above (Figures 2A and 2B) in both sinuses for the presence of a separate origin of coronary arteries. Whenever the opening of the left coronary artery was not identified in Sinus 1, Sinus 2 was inspected for the opening of both coronaries. Usually, in cases of intramural coronary artery, the left coronary artery originates from Sinus 2 at the upper right side of the posterior commissure (Figure 1A). In eight patients, we found that both left and right coronary arteries had an intramural course, arising from Sinus 2 (Figure 2B) with a single opening; one patient had separate openings with kissing edges. The length of the intramural course of the right coronary was nearly 2-3 mm in all cases whereas the length of the intramural course of the left coronary was 4-6 mm (Figure 2C). We used coronary shunts (Synovis Life Technologies, Inc., St. Paul, MN, USA) to probe the coronary artery (Figure 2C) and to find its intramural course in the aortic wall. The posterior commissure was turned down and the stay stitch was placed with a 7-0/8-0 polypropylene suture at the opening of the intramural coronary artery, keeping the coronary shunt in situ (Figure 2D). The entire roof of the intramural coronary arteries was excised (Figure 2F) until the internal opening (Figure 2F) was identified. The bulbous end of the coronary shunt not only helped to identify the internal opening but also prevented any accidental injury to the coronary ostia. A similar procedure was done for the other (right) coronary artery (Figure 2E). Following unroofing, the aortic openings of the coronary ostia were identified in their respective sinuses (Figure 2F). The coronary buttons were excised in routine fashion (Figure 2G) and the proximal epicardial coronary artery was dissected for 2-3 mm to avoid any kink during relocation (Figure 2G). The margins of the coronary buttons were not smooth (Figure 2H) because of the tissue of the posterior commissure. In addition, the buttons of the left coronary were small, but could be comfortably relocated (Figure 2I) to the neo aorta. The left coronary artery was relocated using the punch hole technique in all patients. The right coronary artery was relocated using the trapdoor technique in sixteen patients while the punch hole technique was used for the remaining two patients. The posterior commissure was resuspended after proximal neo pulmonary artery reconstruction. A Lecompte maneuver was performed in all patients with intramural coronary artery. The redundant portion of the proximal neo aorta was plicated posteriorly to form a keel, if required, especially in patients in the TGA/VSD group. The neo aortic and neo pulmonary anastomosis were performed in routine fashion. All patients were weaned off bypass with a moderate dose of inotropes (Adrenaline 0.05 micrograms/kg/ minute and Milrinone 0.5 micrograms/kg/minute) without any difficulty. Mean bypass time was 148 minutes and mean cross-clamp time was 106 minutes in the TGA/IVS group. Mean bypass time was 166 minutes and mean cross-clamp time was 130 minutes in the TGA/VSD group. The chest was left open in eleven patients during the first operative day due to diffused bleeding and it was closed the next day. Postprocedure patients had stable hemodynamic course without any electrocardiogram (ECG) features of myocardial ischaemia.

A- External appearance of the intramural coronary artery.

B- Single ostial opening in sinus 2.

C- Coronary shunt showing the direction of the left intramural coronary artery with a stay stitch on ostial opening.

D- Unroofing of the intramural left coronary artery.

E- Unroofing of the intramural right coronary artery.

F- Unroofed left and right coronary arteries with their ostia in their respective sinuses.

G- Excision of coronary buttons.

H- Neo pulmonary reconstruction using pericardial patch.

I- Coronary relocation to neo aorta.

## RESULTS

For the patient who had an accidental injury to the left coronary artery during dissection, though a 5 mm Gortex (W. L. Gore and Associates, Flagstaff, AZ) graft was used from the innominate artery to the single coronary button for coronary blood supply in a desperate attempt to save him, he died in the operating room secondary to bleeding and severe ventricular dysfunction. One seven-day-old newborn, who underwent TGA with IVS on Prostaglandin infusion and no intraoperative problems, had moderate left ventricular dysfunction on 2D echocardiography six hours after surgery, despite showing stable hemodynamic parameters without any ECG features of myocardial ischaemia. He died from intractable ventricular arrhythmia fifteen hours after surgery. Another patient who had multiple VSD with type-B arch interruption died from residual apical VSD and sepsis on the eleventh postoperative day. The rest of the patients did well in the immediate postoperative period. On every visit, the patients underwent ECG and 2D Doppler echocardiography. One patient had mild valvular pulmonary stenosis with a peak gradient of 20 mmHg and all other patients had patent coronary arteries without any evidence of regional wall motion abnormality, with good biventricular function and no valvular or supra valvular aortic/pulmonary stenosis or regurgitation. So far, none of our patients has undergone myocardial perfusion scan/angiography or any other intervention.

### Survival analysis

**Fig. 3:**
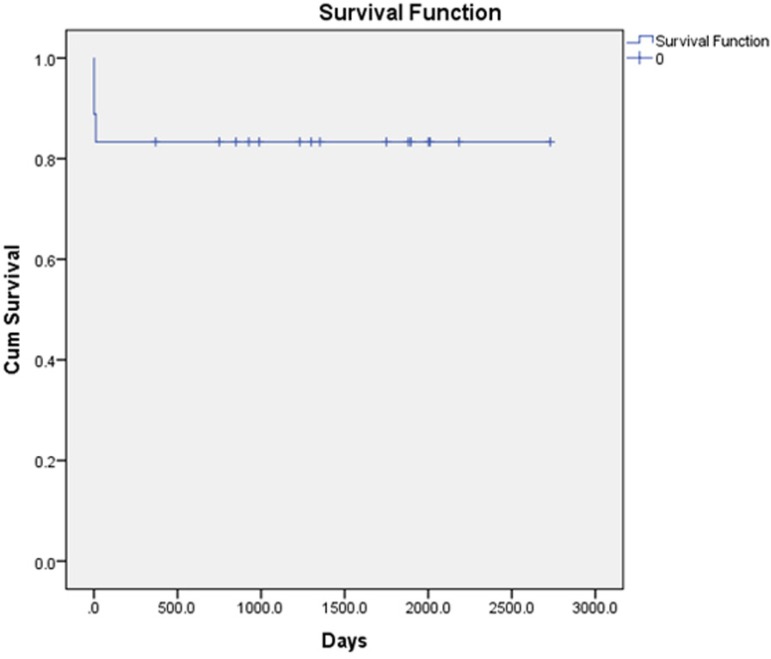
Survival analysis presented in Kaplan-Meier analysis.

The mean follow-up duration was 1235.34±815.26, whereas median was 1788.5 (4.9 years) (range 369 - 2730) days. Out of the 18 patients, overall mortality was observed in 3 (16.66%). The survival analysis was presented as Kaplan-Meier analysis and is shown in Figure 3.

## DISCUSSION

TGA is a common congenital heart disease seen in paediatric cardiac clinics. The ASO is the treatment of choice for patients with TGA. The term "intramural coronary artery" refers to the coronary patterns in which there is an intimate relationship between the aortic and coronary arterial walls; histologically, the aortic and coronary medial walls are attached without interposed adventitia^[2]^. The incidence of intramural coronary artery varies from 1.4% to 5% in various series^[2,3]^. Successful relocation of the coronary arteries is the most important step in ASO. Almost all kinds of coronary patterns can be relocated, however, the presence of an intramural coronary artery offers several surgical challenges and carries high mortality and morbidity^[4-8]^. A meta-analysis by Pasquali et al.^[9]^ reported that the presence of an intramural coronary artery is associated with the highest mortality compared to any other coronary artery pattern, with more than a six-fold increase in mortality compared to the usual coronary arrangement.

Diagnosis of intramural coronary artery in TGA using two-dimensional echocardiography is explained by Pasquini et al.^[10]^. In our experience, intramural coronary artery is always seen as an intraoperative surprise after aortic transection. However, the absence of conical projection of the coronary artery should always raise suspicion in the surgeon's mind. Furthermore, if there is no left coronary artery ostium in sinus one after aortotomy, the surgeon should strongly suspect there is an intramural left coronary artery^[11]^. In our series of patients, we have come across eight patients with a single ostial opening from Sinus 2 (one of them had kissing edges) with intramural course of both left and right coronary arteries, which is very rare^[2,12,13]^. None of the patients in our series had supra commisural origin of intramural coronary artery.

The single button aortocoronary flap technique for intramural coronary artery is associated with a high incidence of coronary complication, especially when the arrangement of the great arteries is anterior and posterior. In that case, the neo pulmonary artery may compress the coronary button and it can cause myocardial ischemia in the immediate postoperative period and, in the long term, there is a possibility of coronary arterial stenosis when the covering tissue does not grow or shrink^[2,11]^. We have no practical experience with the single button technique.

We believe that the creation of two buttons and the transfer of the coronary button to the neo aorta in usual fashion is more physiological, preventing any early or late coronary artery compression and related complications.

The successful surgical treatment for bilateral intramural coronary arteries has been reported earlier^[12,13]^. Our technique of creating two buttons and transferring them in usual fashion is a technical modification of the technique described by Asou et al.^[14]^, and we believe that the incidence of accidental injury to coronary artery is least with our technique.

Our technique of using coronary shunts for identifying the course of the intramural coronary artery and its unroofing is very useful, especially when there is stenosis of the ostium of the left intramural coronary artery. The color of the shunt (usually blue) can be seen (Figure 2C) through the aortic intima and the entire course of the intramural portion of the coronary artery can be delineated. The stay stitch (Figure 2D) provides full control over intimal tissue flap and helps in the unroofing up to the distal portion of the intramural segment. As the coronary shunts are atraumatic and are easily available in various sizes, probing of the intramural coronary artery with metallic coronary probes should be avoided since they may cause trauma to intima as well as dissection and the possibility of late ostial stenosis. In the past, we have also reported the use of coronary shunts for giving ostial cardioplegia^[15]^. The bulbous end of the shunt obliterates the internal opening of the coronary artery and prevents it from any accidental trauma during unroofing while the entire length of the coronary shunt prevents any accidental injury to the tissue around the coronary ostia. We have observed that, with our modified technique, the distal portion of the intramural segment (where the coronary artery actually leaves the aortic wall) can also be opened, which remains potentially stenotic.

## CONCLUSION

ASO is a preferred operation for TGA. However, performing arterial switch in the presence of intramural coronary artery is still challenging in pediatric cardiac surgery, even in experienced hands. Our technique is simple, atraumatic, and it helps to outline the course of intramural coronary arteries, to unroof the intramural coronary artery and to create two buttons. We have observed that, with our technique, ASO in the presence of intramural coronary artery can be performed in routine fashion with acceptable results.

**Table:** 

**Authors' roles & responsibilities**	
AM	Conception and study design; execution of operations and/or trials; analysis and/or data interpretation; final manuscript approval
AJ	Conception and study design; analysis and/or data interpretation; final manuscript approval
MH	Conception and study design; execution of operations and/or trials; statistical analysis; final manuscript approval
VW	Conception and study design; execution of operations and/or trials; final manuscript approval
RP	Conception and study design; execution of operations and/or trials; final manuscript approval
NV	Execution of operations and/or trials; final manuscript approval
DR	Execution of operations and/or trials; final manuscript approval
HP	Execution of operations and/or trials; final manuscript approval
